# P-1757. Longitudinal Wearable Sensor Data Enhance Precision of Long COVID Detection with Machine Learning

**DOI:** 10.1093/ofid/ofaf695.1928

**Published:** 2026-01-11

**Authors:** Chibuike Uwakwe, Ekanath Rangan, Satyajit Kumar, Georg Gutjahr, Xuhui Miao, Emmy Thamakaison, Andrew Brooks, Peter Maguire, Tejaswini Mishra, Lettie McGuire, Michael Snyder

**Affiliations:** Stanford University School of Medicine, Mountain View, CA; Stanford University School of Medicine, Mountain View, CA; Stanford University School of Medicine, Mountain View, CA; Amrita Vishwa Vidyapeetham University, Kochi, Kerala, India; Stanford University, Stanford, California; Stanford University, Stanford, California; Stanford University School of Medicine, Mountain View, CA; Stanford University, Stanford, California; Stanford University School of Medicine, Mountain View, CA; Stanford University School of Medicine, Mountain View, CA; Stanford University School of Medicine, Mountain View, CA

## Abstract

**Background:**

Despite the millions of individuals struggling with persistent symptoms, Long COVID has remained difficult to diagnose due to limited objective biomarkers. Wearable devices are powerful tools for real-time health monitoring through the continuous measurement of objective physiological metrics such as heart rate (HR). Exploring physiological metrics derived from acute SARS-CoV-2 infection periods could provide actionable insights into the progression to Long COVID, ultimately informing management strategies.

**Figure:**
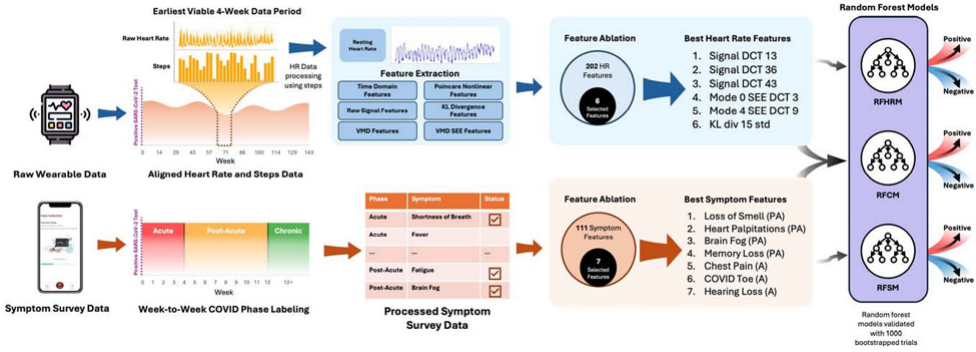
Model Architecture and Data Flow A schematic illustrating the processing pipeline of wearable and symptom data from individuals post-SARS-CoV-2 infection and the architecture of three models: a Random Forest Heart Rate Model (RFHRM), a Random Forest Symptoms Model (RFSM), and a Random Forest Combined Model (RFCM).

**Figure:**
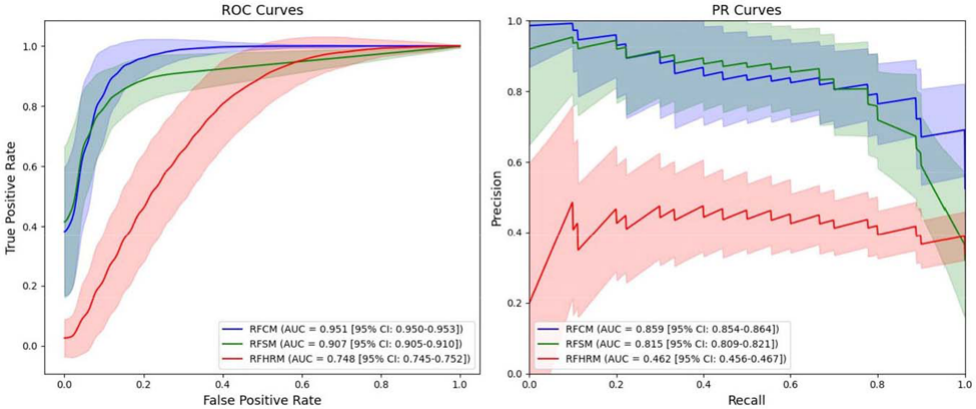
Performance Evaluation of Random Forest Models Average ROC curves and PR curves for the Random Forest Combined Model (RFCM), Random Forest Symptoms Model (RFSM), and Random Forest Heart Rate Model (RFHRM). Error bands indicate the standard deviation of the curves. The legends include the mean AUC for each curve and their corresponding 95% confidence intervals.

**Methods:**

Over 5 years, we collected smartwatch and daily symptom survey data from 126 participants with acute SARS-CoV-2 infections. Those with symptoms > 3 months were labeled as Long COVID. Using acute infection data, we derived features to train three random forest classifiers that identify Long COVID participants: 1) a Heart Rate Model (RFHRM) using only HR features, 2) a Symptoms Model (RFSM) using only symptom features, and 3) a Combined Model (RFCM) using both HR and symptom features. To evaluate the performance of these models, we ran 1,000 bootstrapped trials with 70/30 train-test splits, reinitializing classifiers each time and recording Receiver Operating Characteristic (ROC) and Precision-Recall (PR) curves.

**Figure:**
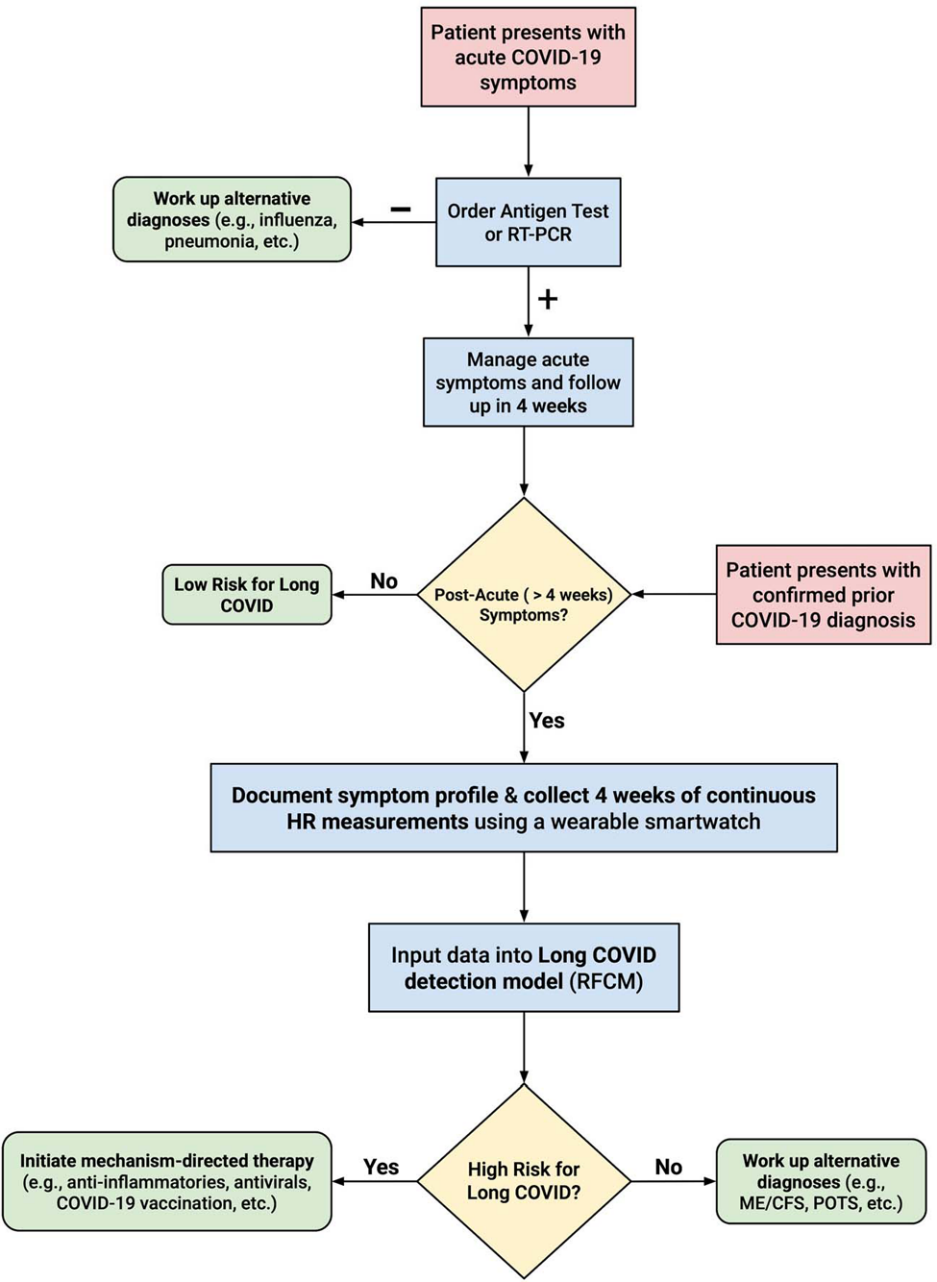
Clinical Use Algorithm Flowchart displaying how a clinician might screen and manage a patient presenting with acute COVID-19 or a confirmed prior COVID-19 diagnosis using our Long COVID detection model.

**Results:**

Of the 126 participants in our study, 31 participants experienced Long COVID symptoms while 95 participants did not. The RFHRM achieved a mean area under the ROC curve (ROC-AUC) of 0.748 (95% CI: 0.745–0.752) and a mean area under the PR curve (PR-AUC) of 0.462 (95% CI: 0.456–0.467). The RFSM achieved a mean ROC-AUC of 0.907 (95% CI: 0.905–0.910) and a mean PR-AUC of 0.815 (95% CI: 0.809–0.821). The RFCM achieved a mean ROC-AUC of 0.951 (95% CI: 0.950–0.953) and a mean PR-AUC of 0.859 (95% CI: 0.854–0.864). These results reflect a statistically significant improvement in Long COVID classification by the RFCM compared to the RFSM.

**Conclusion:**

Our findings provide evidence that non-invasive, wearable-derived metrics can objectively complement clinical histories, potentially transforming existing diagnostic frameworks for Long COVID. The Long COVID detection model we developed could be used to guide clinical management, and decomposing HR data into complex features may aid our understanding of the complex physiology underlying chronic diseases.

**Disclosures:**

Michael Snyder, PhD, Danaher: Advisor/Consultant|GenapSys: Advisor/Consultant|JanuaryAI: Advisor/Consultant|JanuaryAI: Ownership Interest|Jupiter: Advisor/Consultant|Mirvie: Advisor/Consultant|Mirvie: Ownership Interest|Personalis: Advisor/Consultant|Personalis: Ownership Interest|Protos: Advisor/Consultant|Protos: Ownership Interest|Qbio: Advisor/Consultant|Qbio: Ownership Interest|SensOmics: Advisor/Consultant|SensOmics: Ownership Interest

